# Multiplicity of *Plasmodium falciparum *infection in asymptomatic children in Senegal: relation to transmission, age and erythrocyte variants

**DOI:** 10.1186/1475-2875-7-17

**Published:** 2008-01-23

**Authors:** Manijeh Vafa, Marita Troye-Blomberg, Judith Anchang, André Garcia, Florence Migot-Nabias

**Affiliations:** 1 Department of Immunology, Stockholm University, S-106 91 Stockholm, Sweden; 2 University of Buea, Buea, Cameroon; 3 Institut de Recherche pour le Développement (IRD), Unité de Recherche 010 "Santé de la mère et de l'enfant en milieu tropical", BP 1386, Dakar, Senegal; 4 IRD, UR 010, 08 BP 841, Cotonou, Benin

## Abstract

**Background:**

Individuals living in malaria endemic areas generally harbour multiple parasite strains. Multiplicity of infection (MOI) can be an indicator of immune status. However, whether this is good or bad for the development of immunity to malaria, is still a matter of debate. This study aimed to examine the MOI in asymptomatic children between two and ten years of age and to relate it to erythrocyte variants, clinical attacks, transmission levels and other parasitological indexes.

**Methods:**

Study took place in Niakhar area in Senegal, where malaria is mesoendemic and seasonal. Three hundred and seventy two asymptomatic children were included. Sickle-cell trait, G6PD deficiency (A- and Santamaria) and α^+^-thalassaemia (-α^3.7 ^type) were determined using PCR. Multiplicity of *Plasmodium falciparum *infection, i.e. number of concurrent clones, was defined by PCR-based genotyping of the merozoite surface protein-2 (*msp2*), before and at the end of the malaria transmission season. The χ^2^-test, ANOVA, multivariate linear regression and logistic regression statistical tests were used for data analysis.

**Results:**

MOI was significantly higher at the end of transmission season. The majority of PCR positive subjects had multiple infections at both time points (64% before and 87% after the transmission season). MOI did not increase in α-thalassaemic and G6PD mutated children. The ABO system and HbAS did not affect MOI at any time points. No association between MOI and clinical attack was observed. MOI did not vary over age at any time points. There was a significant correlation between MOI and parasite density, as the higher parasite counts increases the probability of having multiple infections.

**Conclusion:**

Taken together our data revealed that α-thalassaemia may have a role in protection against certain parasite strains. The protection against the increase in MOI after the transmission season conferred by G6PD deficiency is probably due to clearance of the malaria parasite at early stages of infection. The ABO system and HbAS are involved in the severity of the disease but do not affect asymptomatic infections. MOI was not age-dependent, in the range of two to ten years, but was correlated with parasite density. However some of these observations need to be confirmed including larger sample size with broader age range and using other *msp2 *genotyping method.

## Background

Multiplicity of infection (MOI) is the number of different *Plasmodium falciparum *strains co-infecting a single host. In malaria endemic areas, MOI can be a useful indicator of the transmission level. Data suggest that the average number of malaria parasite strains in an individual is well correlated to the transmission level [1,2].

MOI can also be an indicator of the immune status. In areas with stable malaria transmission, MOI seems to increase as immunity develops. In asymptomatic children, MOI is suggested to reflect acquired immunity or premunition [3], and also to influence the risk of subsequent malaria attacks. However, several studies have shown an inverse association [4-6] between MOI and malaria attacks, while others have shown a positive correlation between MOI and clinical malaria [7,8]. Thus, it is obvious that the role of MOI for the development of immunity to malaria is still unclear.

A number of molecular epidemiological field studies performed in Papua New Guinea [9], Senegal [10], Tanzania [11], Sudan [1] and Equatorial Guinea [12] has shown that differences in MOI are age-dependent. The age-dependence of MOI is interpreted as a reflection of specific anti-parasite immunity [10]. The age at which efficient immunity is acquired can differ according to transmission intensity; hence, the age of peak MOI could vary amongst different malaria endemic areas.

Erythrocyte variants have been associated with protection against malaria disease. However, the mechanisms behind this protection are not very well understood. Sickle cell trait (HbAS) and α-thalassaemia protect against severe and fatal malaria but none has effect on asymptomatic parasitaemia (Reviewed by Williams TN [13]). Studies on the impact of erythrocyte variants on multiplicity of *P. falciparum *infection are limited and contradictory. Ntoumi *et al *[14] showed that HbAS was associated with high MOI in asymptomatic Gabonese individuals, whereas, no impact of sickle cell trait on MOI was observed in Senegal [15]. Deficiency in the X-linked glucose-6-phosphate dehydrogenase (G6PD) gene reduces the risk of developing severe malaria. The most common G6PD deficiency in sub-Saharan Africa is G6PD A- [16], which is believed to have been selected by the malaria parasite [17]. Similarly, ABO blood group antigens have been correlated with severe pathology, where blood group O confers protection against malaria, in contrast to blood group A and B [18]. The impact of G6PD deficiency and ABO blood group on multiplicity of *P. falciparum *infection has so far not been investigated.

The merozoite surface proteins (MSP) are involved in erythrocyte invasion [19] and affect parasite density and eventually severe pathology. Genotyping of *msp2 *is a standard method for assessing MOI [20], as it is highly polymorphic in length and sequence [10].

This study examined; i) the influence of erythrocyte variants on MOI; ii) the association of MOI with the risk of developing clinical symptoms and iii) the correlation of MOI with age, parasite density and seasonal variation, in asymptomatic children living in a malaria mesoendemic area in Senegal. Results of this study indicated that both α-thalassaemia and G6PD deficiency affected the seasonal variation of MOI. However, MOI was not influenced by the ABO system and HbAS. MOI was not dependent on age (in the range of two to ten years), was correlated with parasite density, increased after the transmission season and was not associated with the risk of clinical attack.

## Methods

### Study area and subjects

The study took place in two villages (Diohine and Toucar) in the Niakhar area, 115 km south-east of Dakar, Senegal. Malaria is mesoendemic in this area and transmission is seasonal from September to December, with an average of 9–12 infective bites per person and year [21]. A cohort of 372 unrelated children aged from 2 to 10 years, living permanently in the area was included in this study.

### Sample collection

Twenty microlitres of blood were collected on filter paper in June 2002 and January 2003, i.e. before and after the malaria transmission season. Venous blood was collected into heparinized Vacutainer^® ^tubes (Becton Dickinson, Meylan, France) at initiation of study in June 2002. Parasitological measurements were performed from thick blood smears (TBS) obtained by finger prick at both time points. After staining with Giemsa, leucocytes were counted in 30 standardized microscopic fields in order to obtain a mean number of leucocytes per field, and parasites were counted on 50 fields in order to define the parasitaemia as the number of parasites per 100 leucocytes. This mode of calculation allowed us to avoid under- or over-estimations of the parasitaemia resulting from its determination on the basis of an assumed count of white blood cells per microliter of blood [22]. A TBS was declared negative when no parasite was detected in 200 fields. Research was carried out in compliance with the Helsinki Declaration. Parents of all children gave written informed consent, and the protocol was approved by the ethics committee of the Health Ministry of Senegal (N°000526/MS/DERF/DER).

### Clinical survey

Part of the material was used in a malaria genetic epidemiology programme. In this study an active survey of malaria attacks was conducted during the transmission period for a sub group of 154 children corresponding to the youngest children from each nuclear family [23] and this sub group was also included in present study. Axillary temperature was measured twice a week, and a thick blood smear was done in the case of fever (axillary temperature higher than 37.5°C) or history of fever. A malaria attack was defined as the association of fever with a parasite density higher than 2500/μl without any other apparent cause of fever. Morbidity data generated a qualitative variable according to the absence or the presence of at least one malaria attack during the transmission season. During the whole study, all children had free access to the dispensary for diagnosis and treatment and all information was registered.

### Human genotyping

Genomic DNA was extracted using an automaton system (Auto Gen NA 2000^®^). ABO blood groups, sickle-cell trait, G6PD deficiency (A- and Santamaria) and α^+^-halassaemia (-α^3.7 ^type) were already determined as previously published [24].

### Parasite genotyping

DNA was extracted from blood on filter papers. Discs of the same size (half of the dried blood blot) were cut out and incubated overnight in 1 ml of 0.5% saponin in phosphate buffered saline (PBS) at 4°C. Discs were boiled in 200 μl of 5% Chelex-100 in water for 15 minutes after being washed 15–30 minutes in PBS at 4°C. After centrifugation at 10,000 rpm for three minutes, DNA containing supernatant was collected. Nested PCR was performed to determine the numbers of *msp2 *(FC27 and 3D7) clones. Amplifications were done in 10 μl reaction mixture containing DNA template, iProof™ High-Fidelity Master Mix (BIO-RAD Laboratories, Hercules, CA) and 500 nM of primer pairs. A primer pair corresponding to the outer conserved region of the polymorphic repetitive block 3 of *msp2 *was used to amplify 2.5 μl of DNA extracted from filter paper. DNA was denatured at 98°C for one minute; then PCR was performed for 30 cycles of 98°C/10 sec, 61°C/20 sec, 72°C/30 sec, with a final five-minute extension. One microliter of PCR product was reamplified in a pair of reactions using primers specific for FC27 and IC/3D7 allelic types of *msp2*, using following program: 30 sec at 98°C; 25 cycles of 98°C/10 sec, 61°C/20 sec, 72°C/10 sec; and a final extension for five minutes. The sequences of used primers have been presented elsewhere [25]. The number of products, corresponding to number of infecting FC27 and IC/3D7 clones, was counted after visualization on Ethidium Bromide (EtBr) stained 2.5 and 1.5% agarose gels, respectively.

### Statistical analysis

The χ^2^-test was used to compare qualitative variables such as parasite prevalence and malaria morbidity between different groups or time points. Analyses of positive parasite densities were conducted after a logarithmic transformation. Univariate analyses of quantitative variables such as log-transformed parasite density or number of clones were performed using ANOVA. However, in case of unequal variances (Bartlett's Test) non-parametric statistic was used. Furthermore, multivariate linear regression was used to test for confounding and independent associations between age, parasite density and MOI. A logistic regression was used to test the association between malaria morbidity (occurrence of malaria attack), MOI and age. *P *values of <0.05 were considered to indicate statistical significance. As our objective was mainly to generate hypotheses on the putative role of MOI on the protection conferred by erythrocyte variants, we did not apply corrections for multiple testing when we studied the potential association between red blood cells polymorphisms and MOI.

All statistical analyses were performed using StatView 5.0 (SAS Institute Inc., Cary, NC) and STATA (StataCorp. 1999, Release 8.0) softwares.

## Results

### Parasitological indexes

A total of 372 children in the age range of 2 to 10 years (5.0 ± 1.6) was included in this study. Parasitological indexes are presented in Table 1. Asymptomatic *P. falciparum *prevalence was detected by microscopy in 35% of individuals both before (June 2002) and after (January 2003) the transmission season. Parasite prevalence, as detected by PCR, was higher in January as compared to June, although the difference did not reach statistical significance.

**Table 1:** Parasitological indexes before and after transmission season

	June 2002	January 2003	*P *value
	
Age-range (years)	2–10	2–10	
Mean age ± SD	5.0 ± 1.6	5.6 ± 1.6	
Sex ratio (M/F)	1.1 (195/177)	1.1 (195/177)	
Parasite prevalence^a ^(%)			
Microscopy	35	35	
PCR	44	51	NS
Parasite density^b^	2.9	7.6	0.01
Parasite density 25th–75th percentiles	0.8–11.2	1.3–33.1	
MOI^c ^(mean)	2.5	4.1	0.0001
MOI (range)	1–7	1–10	
Number of subjects	372	372	

^a ^Proportion of infected individuals.

^b ^*P. falciparum *density (parasites/100 leukocytes)-median, zero values excluded.

^c ^Number of clones identified as *msp2 *alleles, excluding PCR negatives.

The median parasite density was lower before (2.9 [0.8–11.2]/100 leukocytes) than after (7.6 [1.3–33.1]/100 leukocytes) the transmission season (rho = 0.36, *P *= 0.01). Interestingly, for a small group of 58 cases, infected at both time points, no increase in parasite density was seen after the transmission season (not listed).

Before the transmission season, parasite density decreased significantly with age (regression coefficient = -0.36; *P *= 0.004) when subjects were grouped into 2, 3, 4, 5 and 6–10 years, whereas the effect of age on PD was no more significant in January (*P *> 0.18).

No significant differences in the prevalence of infection, as detected by PCR, amongst different age groups (grouped as above) were noted at any time points.

### Multiplicity of Infection

MOI has been suggested to differ in relation to transmission intensity, age, parasite density and seasonal variation. Therefore, the MOI before and after transmission and its relationship with the above mentioned factors was investigated in this study. The results revealed that the MOI (defined on PCR positive samples) was significantly higher after the transmission season (mean 4.1 [1-10] strains in January vs 2.5 [1-7] strains in June, *P *< 0.0001) (Table 1). The majority of PCR positive subjects carried multiple strains; 64 and 87 percent in June and January, respectively.

The distribution of *msp2 *allelic families was similar before and after transmission season, with 35% (33%) of parasites from 3D7, 25% (24%) of parasites from FC27 and 40% (43%) of clones from both allelic families in June (January).

There was no significant association between age and MOI in June (*P *> 0.1) or in January (*P *> 0.7). MOI was positively related to parasite density at both time points (*P *= 0.004 in June and *P *< 10^-3 ^in January). However, as age was negatively related to PD, a multiple linear regression including age as an explicative variable was performed. Age remained no significant (*P *> 0.5) and the same pattern of results was obtained at each time points consistent with a positive association between MOI and PD (*P *= 0.008 in June and *P *< 10^-3 ^in January).

### Parasitological data in relation to erythrocyte variants

Erythrocyte variants may alter susceptibility to parasite invasion and thus affect the multiplicity of infection. This study revealed that the blood groups or the studied RBC mutations did not influence the MOI at any time points (Table 2). After the transmission season, the MOI increased significantly in all groups of individuals except the alpha-thalassaemic and the G6PD mutated cases.

**Table 2:** Infection prevalence and MOI in subjects stratified by blood groups and haemoglobinopathies before and after the transmission season

		June 2002		January 2003	
		
	Whole group^a ^n (%)	n^b ^(%)	MOI; mean (range)	n^b ^(%)	MOI; mean (range)
Blood group					
O	193 (52)	81/193 (42)	2.6 (1 – 7)	99/193 (51)	4.1 (1–10)^g^***
A	100 (27)	47/100 (47)	2.6 (1 – 7)	50/100 (50)	4.0 (1 – 9)^g^**
B	69 (18)	34/69 (49)	2.3 (1 – 6)	32/69 (46)	4.2 (1 – 10)^g^*
AB	10 (3)	2/10 (20)	4.0 (1 – 7)	7/10 (70)	4.6 (2 – 8)^g^*
Hemoglobin					
AA	319 (86)	144/319 (45)	2.5 (1 – 7)	162/319 (51)	4.0 (1–10)^g^***
AS	51 (14)	19/51 (37)	2.7 (1 – 5)	26/51 (51)	4.5 (1 – 10)^g^**
α^+^-thalassemia^C^					
Normal	234 (76)	103/234 (44)	2.3 (1 – 7)	118/234 (50)	3.9 (1–10)^g^***
Mutation	75 (24)	37/75 (49)	2.8 (1 – 7)	36/75 (48)	4.1 (1 – 9)
G6PD in females^d^					
Normal	141 (81)	60/141 (43)	2.7 (1 – 7)	73/141 (52)^f^	4.4 (1 – 10)^g^**
Mutation	34 (19)	11/34 (32)	2.7 (1 – 4)	9/34 (26)	4.6 (1 – 10)
G6PD in males^e^					
Normal	166 (87)	77/166 (46)	2.5 (1 – 7)	90/166 (54)	3.8 (1–10)^g^***
Mutation	24 (13)	14/24 (58)	1.7 (1 – 4)	14/24 (58)	3.4 (1 – 7)

^a ^Total number of samples = 372, missing values for hemoglobin (n = 2), α^+^-thalassemia (n = 63) and G6PD (n = 7).

^b ^Proportion of infected individuals to total.

^c ^Mutation indicated in both heterozygous and homozygous state.

^d ^G6PD mutations include G6PD A-/B, A-/A heterozygous and A-/A- homozygous genotypes.

^e ^G6PD mutation corresponds to A- hemizygous genotype.

^f ^Significantly less infection prevalence in G6PD mutated girls as compared to normal ones; χ^2^-test, P < 0.01.

^g ^Wilcoxon signed rank test for the comparison of MOI in June and January;

* *P *< 0.05

** *P *< 0.01

*** *P *< 0.0001

**Figure 1:**
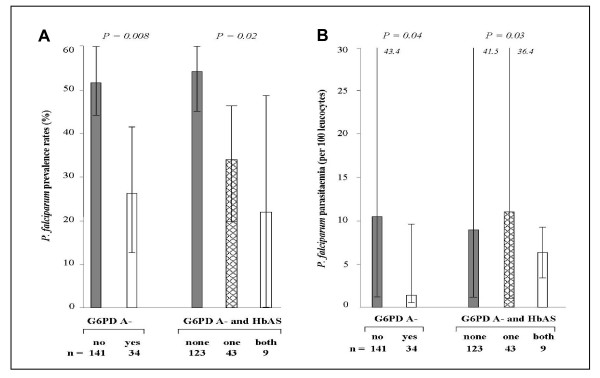
**Parasite prevalence (A) and densities (B) amongst girls carrying G6PD A- or G6PD A-/HbAS variants**. Parasite prevalence was defined as the percentage (CI 95%) of infected subjects detected by PCR. Parasite densities were compared as median (25th–75th percentiles) excluding zero values.

At the end of the transmission season, in G6PD mutated girls, the parasite prevalence (*P *= 0.008, Figure 1A) and density (*P *= 0.04, Figure 1B) were significantly reduced as compared to the girls carrying the normal gene.

**Figure 2:**
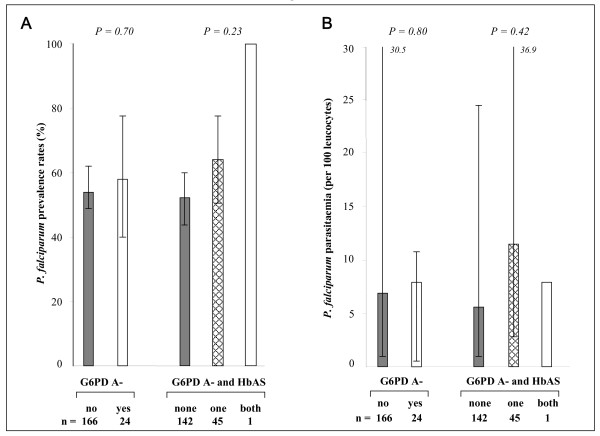
**Parasite prevalence (A) and densities (B) amongst boys carrying G6PD A- or G6PD A-/HbAS variants**. Parasite prevalence was defined as the percentage (CI 95%) of infected subjects detected by PCR. Parasite densities were compared as median (25th–75th percentiles) excluding zero values.

G6PD A- girls carrying the sickle cell trait (n = 9) had significantly lower *P. falciparum *prevalence (*P *= 0.02, Figure 1A) and counts (*P *= 0.03, Figure 1B) as compared to single mutated girls (AS or G6PD A-, n = 43) and girls carrying neither of these genetic traits (n = 123) (Figures 1A and 1B). Inversely, these erythrocyte variants had no effect on parasitological data obtained for boys (Figures 2A and 2B).

Parasite prevalence (*P *= 0.05) and number of concurrent clones (*P *= 0.05) were to borderline to be significantly lower, in alpha-thalassaemic children with sickle cell trait (n = 10) as compared to the others (n = 298).

### Correlation between MOI and malaria morbidity

MOI has been shown to influence clinical manifestation; hence the association between MOI and malaria attacks was examined in present study. Seventy (45%) out of 154 children for whom the clinical survey was performed had a malaria attack. Of children who had at least one malaria attack, 57 and 84 percent had multiple infections in June and January, respectively. However, no significant association was observed between MOI and clinical presentation (OR = 1.15; IC95 = [0.55–2.41]; *P *> 0.7).

## Discussion

Multiplicity of malaria infection is suggested to be influenced by age, transmission intensity and seasonal variation [3,15,26,27]. As expected, a general higher parasite density and MOI after the transmission season was observed in this study.

The distribution of *msp2 *3D7/FC27 allelic families before (40/40 percent) and after (43/44 percent) the transmission season was similar. These figures are very close to those reported in Gambia [28], Papua New Guinea [9] and Senegal [29] but differ from those observed in other areas (differing in malaria transmission intensity) in Senegal [10,30]. This suggests that heterogeneity of *P. falciparum *infection may differ according to geographical location, transmission intensity and season of sampling.

As has been proposed by previous studies [31,32], high parasite densities increase the probability of detecting concurrent clones in an individual. Consistent with this, a positive correlation between MOI and parasite density was observed in this study.

Whether or not MOI is influenced by RBC variants was examined by present study. ABO blood group had no impact on MOI at any time points. This probably reflects the fact that the ABO system does not affect the parasite densities rather the clinical outcome of the disease, where blood group O protects against cerebral malaria [18].

Previous studies on the association between sickle cell trait and MOI are contradictory [14,15]. No effect of HbAS on MOI was noted in present study. This is in line with reports by Konate *et al *[15] in Senegal but in contrast to what has been shown in Gabon [14]. The reasons for these discrepancies are probably due to differences in the transmission intensity and in the ages of the study groups.

Alpha-thalassaemic red blood cells have altered membrane properties [33,34], which may affect erythrocyte invasion by merozoites [19]. MSP2 antigen is involved in invasion [19] and is polymorphic. Hence, changes in membrane of α-thalassaemic RBCs may interrupt invasion by parsites with certain *msp2 *variants, and that could eventually influence the MOI. In accordance with this, no significant increase in the MOI, after the transmission, was seen in the α-thalassaemic children. This suggests that α-thalassaemic RBCs may protect against invasion by certain parasite strains. However, these data contradicts findings reported by Mockenhaupt *et al *[35], who did not find an influence of α-thalassaemia on the prevalence, density and multiplicity of symptomatic *P. falciparum *infection. It is now evident that α-thalassaemia does not affect parasite density in individuals that go on to present clinical malaria [35-37]. The reason for these discrepancies might be due to that Mockenhaupt *et al *studied symptomatic patients, while present study comprised asymptomatic children. Differences in sample sizes of the two studies could also be a reason for contradictory observations. In any case these data indicate that α-thalassaemic individuals may be less susceptible to infection by certain parasite strains but once they get infected they are not protected against clinical malaria.

Similar to α-thalassaemic, G6PD mutated children did not show a significant increase in the MOI after the transmission season. This may be explained by early phagocytosis of infected G6PD deficient RBCs. Cappadoro *et al *[38] have shown that ring-stage infected G6PD deficient RBCs are 2.3 times more efficiently phagocytosed than normal iRBC. Thus, G6PD mutated individuals probably control the parasite growth better than subjects carrying the normal G6PD gene. This would then lead to lower MOI as a result of lower parasite counts at the end of transmission season. This is supported by the fact that girls with mutated G6PD, in this study, were found to have significantly lower parasite prevalence and density as compared to the girls carrying the normal gene. This phenomenon was reinforced in the case of girls carrying G6PD A- and HbAS combined erythrocyte defects, whereas HbAS alone was not found to influence MOI over the transmission season. No similar observation could be made for boys, for whom the G6PD A- carriage at the hemizygous status is assuredly responsible for enzyme deficiency: the small sample size of the groups of erythrocyte variant (considered alone or in combination) carriers, could have generated this absence of divergent parasite data between groups of males.

Previous studies regarding the variation of MOI over age have suggested that the influence of age on the multiplicity of infection is highly affected by endemicity of malaria [1,9-11]. This is probably a reflection of the development of anti-parasite specific immunity [10]. Thus, in holo- or hyperendemic area, immunity develops faster and at younger age than in areas with less intense transmission [39]. Studies have shown an age-dependent MOI in a village with intense perennial malaria transmission but not in areas where malaria is mesoendemic [15,29]. In line with this, here we showed that MOI is not influenced by age, at least not in the age range of 2 to 10 years. However, the lack of correlation between MOI and age in present study needs to be confirmed in a study population with broader age range.

Reports regarding the relation of MOI and malaria morbidity are contradictory. A number of studies has suggested that high MOI may confer protection from subsequent clinical malaria [4-6]. A few studies also have associated malaria morbidity with high MOI [7,8]. In contrast with those, no correlation between MOI and the risk of clinical presentation was noted in this study. This contradiction may be in part due to variable genotyping protocols, differing in sensitivity and accuracy, used in different laboratories [15,32,40,41]. In this study, sampling was done only at two time points and MOI was not determined at the time of clinical presentation. This might be another possible reason for the lack of correlation between MOI and development of clinical malaria, noted in the present study. Moreover, this result underlines the fact that parasite characteristics are certainly not the only ones involved in malaria morbidity. Host characteristics such as genetic polymorphisms, or transmission intensity could also play a key role.

However, to be able to compare findings by different studies on diversity of *P. falciparum *infection in relation to other parasitological indexes and/or host factors, performed in different areas, methodology used by these studies should be taken into consideration.

The genotyping results generated by PCR may differ between different laboratories because of applying variable reagents and protocols or even handling [32]. In addition, the PCR used in this study to determine diversity of parasite population do not discriminate between virulent and avirulent strains. This could perhaps be a general explanation for some of our observations here such as the lack of association between MOI and malaria morbidity, or the variable effects of erythrocyte variants on parasite diversity and clinical pattern of the disease.

## Conclusion

In summary, results of this study suggest that; α-thalassaemia may protect against infection by certain *P. falciparum *strains, the protection conferred by G6PD deficiency is probably through clearance of the malaria parasite at early stages of infection, and that the ABO system and HbAS do not affect asymptomatic infection. These data also revealed that the multiplicity of *P. falciparum *infection in asymptomatic children living in a mesoendemic area was correlated with parasite density but not age, in the range between two to ten years. MOI did not influence the risk of clinical attack. However, to be able to draw a firm conclusion, this needs to be repeated and confirmed by others and/or using other methodology. Larger sample size and broader age range are also required to confirm observations by this study.

## Authors' contributions

MV performed parasite genotyping, participated in DNA extraction from filter papers and drafted the manuscript. MTB participated in study design and coordination and revised the manuscript. JA contributed to DNA extraction for parasite genotyping. AG performed the statistical analysis, helped to draft the results and revised the manuscript. FMN conceived of the study, participated in study design and coordination, carried out human genotyping, helped in statistical analysis and revised the manuscript. All authors read and approved the manuscript.
